# Can Variation in Hypothalamic-Pituitary-Adrenal (HPA)-Axis Activity Explain the Relationship between Depression and Cognition in Bipolar Patients?

**DOI:** 10.1371/journal.pone.0037119

**Published:** 2012-05-14

**Authors:** Marieke J. van der Werf-Eldering, Rixt F. Riemersma-van der Lek, Huibert Burger, Esther A. E. Holthausen, André Aleman, Willem A. Nolen

**Affiliations:** 1 Department of Psychiatry, University Medical Centre Groningen, University of Groningen, Groningen, The Netherlands; 2 Department of Epidemiology, University Medical Centre Groningen, University of Groningen, Groningen, The Netherlands; 3 BCN Neuroimaging Centre, University of Groningen, Groningen, The Netherlands; Baylor College of Medicine, United States of America

## Abstract

**Background:**

Dysregulation of the hypothalamic-pituitary-adrenal (HPA) axis is thought to be associated with more mood symptoms and worse cognitive functioning. This study examined whether variation in HPA axis activity underlies the association between mood symptoms and cognitive functioning.

**Methodology/Principal Findings:**

In 65 bipolar patients cognitive functioning was measured in domains of psychomotor speed, speed of information processing, attentional switching, verbal memory, visual memory, executive functioning and an overall mean score. Severity of depression was assessed by the Inventory of Depressive Symptomatology-self rating version. Saliva cortisol measurements were performed to calculate HPA axis indicators: cortisol awakening response, diurnal slope, the evening cortisol level and the cortisol suppression on the dexamethasone suppression test. Regression analyses of depressive symptoms and cognitive functioning on each HPA axis indicator were performed. In addition we calculated percentages explanation of the association between depressive symptoms and cognition by HPA axis indicators. Depressive symptoms were associated with dysfunction in psychomotor speed, attentional switching and the mean score, as well as with attenuation in diurnal slope value. No association was found between HPA axis activity and cognitive functioning and HPA axis activity did not explain the associations between depressive symptoms and cognition.

**Conclusions/Significance:**

As our study is the first one in this field specific for bipolar patients and changes in HPA-axis activity did not seem to explain the association between severity of depressive symptoms and cognitive functioning in bipolar patients, future studies are needed to evaluate other factors that might explain this relationship.

## Introduction

Bipolar disorder is characterized by recurrent (hypo)manic and depressive mood episodes. Moreover, many, but not all bipolar patients show cognitive dysfunction in areas of attention, memory and executive functioning, not only during mood episodes [1], [2] but also when euthymic [3], [4] possibly indicating trait-like expressions of a genetic phenotype. However, cognitive dysfunction may be more pronounced during depressive episodes [1], [2].

Dysregulation of the hypothalamic-pituitary-adrenal (HPA) axis has been suggested to be involved [5]–[7]. For many years a change in set point of the HPA system, resulting in altered regulation of cortisol secretory activity [ultimately expressed as hypercortisolism and impaired glucocorticoid receptor function; [8]] has been emphasized in (unipolar) depression [9]–[11] as well as bipolar disorder [12]–[14]. Moreover, patients with more severe illness characteristics, such as longer duration of illness and higher number of episodes, as well as psychotic features, appear to show even worse HPA dysregulations [13]–[16].

All these characteristics are difficult to disentangle, but they are considered relevant in the pathogenesis and pathophysiology of bipolar disorders; endocrine abnormalities are thought to lead not only to affective symptoms, but also to contribute to cognitive problems due to partly reversible structural changes in the brain [7], [13], [17]. Patient and illness characteristics such as age and severity of the illness have been suggested as determinants of these problems. Although the underlying mechanism is essentially unknown, dysregulation of the HPA-axis is thought to be involved. Although the precise time course remains unclear, fast and reversible negative effects of cortisol on cognition have been demonstrated in animal and human model studies [18]. Studies in healthy younger [19] and older [20] populations suggested that psychosocial stress and subsequent cortisol excess is one of the mechanisms underlying (reversible) cognitive dysfunction. Regarding depression, many studies indicated an important role for HPA axis hyperactivity (including the use of corticosteroids) in its pathophysiology, affecting cognition through different neuronal networks [6], [7], [13], [15], [17], [21], [22]. Consequently, HPA axis hyperactivity has been the focus of novel therapeutic approaches targeting the HPA axis in order to improve not only mood symptoms, but also cognitive functioning [6], [15], [17].

**Table 1:** Data of 14 cognitive outcome variables, summarized in 6 domains (bold) for 65 bipolar patients.

	Raw data	
	Mean	SD
**Psychomotor speed**		
Simple movement time (msec)	450.06	133.77
Five-choice movement time (msec)	409.45	109.24
**Speed of information processing**		
Simple reaction time (msec)	349.64	95.33
Five-choice reaction time (msec)	383.65	103.94
Stroop time 1 (word; sec)	44.49	8.67
Stroop time 2 (color; sec)	57.74	12.84
**Attentional switching**		
Difference CPT hitrate version Q minus HQ	0.05	0.07
- CPT hitrate version Q (% correct)	0.996	0.01
- CPT hitrate version HQ (% correct)	0.947	0.07
**Verbal memory**		
CVLT – verbal learning (total nr correct resp)$	53.94	12.09
CVLT – long term free recall (nr correct resp)$	12.25	3.18
**Visual memory**		
PRM – number correct immediate$	10.57	1.54
PRM – number correct delayed$	9.34	1.85
**Executive functioning/working memory**		
SWM – between errors 8 boxes (nr correct resp)	23.46	12.23
SWM – strategy (efficiency score)	35.37	5.22
Stroop interference (difference rate; sec)	7.22	22.14

$ higher score indicates better performance; in further analyses age-adjusted data were reversed (100 minus score).

CPT: Continuous Performance Task; CVLT: Dutch version of California Verbal Learning Test; PRM: Pattern Recognition Memory; SWM: Spatial Working Memory; msec: milliseconds; sec: seconds; nr: number; resp: responses.

The first studies to disentangle the association between mood symptoms, HPA responses and cognitive functioning have shown different results. Reppermund et al. [23] studied 75 inpatients with major depressive disorder and revealed that slowed speed of information processing measured by averaging the time of two matrices of the Zahlenverbindungstest was associated with more mood symptoms, whereas improvement of verbal short term memory was associated with decreased cortisol response, which was evaluated with the dexamethasone (DEX)/corticotropin-releasing hormone (CRH) test. Egeland et al. [24] studied 26 patients with major depressive disorder and found that impairments in executive functioning and memory retrieval, both typical for depressive patients, were related to morning cortisol levels, whereas psychomotor retardation was associated with severity of mood symptoms. Zobel et al. [25] studied 64 patients with major depressive disorder and described an improvement of working memory instead of depressive symptoms after normalization of cortisol response pattern in the DEX/CRH test; no relationship was found between episodic memory and cortisol.

**Table 2:** Characteristics for the 65 bipolar patients.

	Mean	SD
Age (years)	46.9	10.4
Premorbid IQ	107.5	9.3
Education level (range 1–6)	3.6	1.0
Duration of illness (years)	21.8	13.1
IDS-SR	17.0	10.7
YMRS	0.35	1.1
Cortisol (mmol/dL)		
- T1, at awakening	16.8	6.8
- T2, 30 min after awakening (n = 63)	18.8	8.4
- T3, 45 min after awakening (n = 61)	19.5	10.8
- T4, 60 min after awakening (n = 62)	18.4	12.8
- AUCg (n = 61)	18.4	7.5
- AUCi (n = 61)	1.8	7.0
- T5, at 10 PM (n = 59)	4.4	2.7
- T6, at 11 PM (n = 62)	5.2	4.6
- mean evening cortisol (n = 58)	4.8	3.1
- diurnal slope decline per hour (n = 62)	0.7	0.6
- T7, at awakening (n = 56)	7.3	3.7
- DST suppression ratio (n = 56)	3.1	2.7

	N	%
Female gender	44	67.7
Bipolar disorder type I	53	81.5
Type of medication		
- Lithium	43	66.2
- Anticonvulsants‡	26	40.0
- Antipsychotics	15	23.1
- Antidepressants	10	15.4
- Benzodiazepines	5	7.7
- None	2	3.1

‡ 1 patient used two different types of anticonvulsants.

AUCg: area under the curve (AUC) with respect to the ground; AUCi: AUC with respect to the increase; DST: cortisol suppression on the dexamethasone suppression test; IDS-SR: Inventory of Depressive Symptomatology-self rating; YMRS: Young Mania Rating Scale.

### Aim of the study

The interplay between mood symptoms and HPA axis activity in their relation with cognitive functioning in bipolar patients is unclear. The present study is, to the best of our knowledge, the first to investigate the HPA axis as a factor explaining the association between depressive symptoms and cognitive functioning in bipolar disorder. Based on the sparse literature as described above, we expected to find an increased HPA axis activity in patients with cognitive dysfunction. As we have found in a previous study that cognitive functioning was associated with depressive symptoms [26], we hypothesized that HPA axis activity might explain this association.

**Table 3:** Associations between depressive symptoms and cognitive performance, presented in subgroups of availability of the respective HPA indicator.

	CAR (n = 61)	Evening (n = 58)	Diurnal (n = 62)	DST (n = 56)
	Beta† [95%CI]	Beta† [95%CI]	Beta† [95%CI]	Beta† [95%CI]
Speed	0.45 [0.12; 0.78]	0.50 [0.17; 0.83]	0.47 [0.14; 0.79]	0.47[0.16; 0.78]
Process	0.35 [−0.01; 0.70]	0.26 [−0.11; 0.63]	0.26 [−0.10; 0.61]	0.29 [−0.06; 0.65]
Attention	0.28 [0.05; 0.51]	0.25 [0.01; 0.48]	0.26 [0.04; 0.49]	0.02 [0.06; 0.53]
Verbal	0.003 [−0.32; 0.33]	0.02 [−0.29; 0.34]	0.08 [−0.24; 0.40]	0.03 [−0.30; 0.36]
Visual	0.10 [−0.17; 0.36]	0.13 [−0.13; 0.40]	0.14 [−0.12; 0.40]	0.09 [−0.17; 0.34]
Exec/WM	0.02 [−0.26; 0.30]	0.02 [−0.26; 0.31]	0.03 [−0.24; 0.29]	−0.01 [−0.29; 0.27]
Mean	0.20 [0.03; 0.36]	0.20 [0.02; 0.37]	0.21 [0.04; 0.37]	0.19 [0.03; 0.36]

† All values are regression coefficients (beta's) indicating the mean change in z-score of cognitive performance, associated with an increase of 13 points IDS-SR score.

Higher values indicate worse performance.

Betas are corrected for age, gender, education and IQ.

Speed = psychomotor speed; Process = speed of information processing; Attention = attentional switching; Verbal = verbal memory; Visual = visual memory; Exec/WM = executive functioning/working memory; Mean = mean z-score of all 6 cognitive domains; CAR = cortisol awakening response; evening = mean evening cortisol level; Diurnal = diurnal slope; DST = cortisol suppression on the dexamethasone suppression test; 95%CI = 95% confidence interval.

## Materials and Methods

### Ethics Statement

The procedures were approved by the institutional review board of the University Medical Center Groningen (reference numbers METc2005.236 and METc2007.200), and in accordance with the latest version of the Declaration of Helsinki. All participants had the capacity to consent, since strict inclusion and exclusion criteria were followed with regard to patient characteristics which could be able leading to reduced capacity to consent (e.g. excluding those patients suffering from severe depressive symptoms and/or (hypo)manic symptoms or patients with IQ<70; for further details see section 2.2). All participants gave written informed consent after the nature of the procedures had been fully explained.

**Table 4:** Associations between HPA categories and cognitive performance.

	CAR (n = 61)	Evening (n = 58)	Diurnal (n = 62)	DST (n = 56)
	Beta† [95%CI]	Beta† [95%CI]	Beta† [95%CI]	Beta† [95%CI]
Speed	−0.03 [−0.06; 0.01]	0.03 [−0.07; 0.12]	0.03 [−0.47; 0.53]	0.09 [−0.01; 0.19]
Process	0.01 [−0.03; 0.05]	0.01 [−0.09; 0.11]	0.09 [−0.43; 0.60]	0.06 [−0.05; 0.18]
Attention	0.02 [−0.01; 0.04]	−0.01 [−0.08; 0.05]	−0.05 [−0.39; 0.29]	0.05 [−0.02; 0.13]
Verbal	−0.01 [−0.05; 0.02]	−0.03 [−0.11; 0.05]	−0.39 [−0.83; 0.06]	−0.04 [−0.14; 0.06]
Visual	0.02 [−0.01; 0.05]	0.004 [−0.07; 0.07]	0.08 [−0.29; 0.46]	−0.01 [−0.09; 0.08]
Exec/WM	0.02 [−0.01; 0.05]	0.01 [−0.06; 0.09]	−0.28 [−0.66; 0.10]	0.03 [−0.06; 0.11]
Mean	0.003 [−0.02; 0.02]	0.001 [−0.05; 0.05]	−0.09 [−0.34; 0.16]	0.03 [−0.02; 0.09]

† All values are regression coefficients (beta's) indicating the mean change in cognitive performance, associated with an increase of 1 unit HPA value.

Higher values indicate worse performance.

Betas are corrected for age, gender, education and IQ.

Speed = psychomotor speed; Process = speed of information processing; Attention = attentional switching; Verbal = verbal memory; Visual = visual memory; Exec/WM = executive functioning/working memory; Mean = mean z-score of all 6 cognitive domains; CAR = cortisol awakening response; evening = mean evening cortisol level; Diurnal = diurnal slope; DST = cortisol suppression on the dexamethasone suppression test; 95%CI = 95% confidence interval.

### Participants

The current study was part of a larger study in bipolar patients we performed between October 2005 and December 2008 in which we explored the kind and extent of cognitive functioning as well as the association with depressive symptoms [26]. All patients (age 18–65 years) had DSM-IV bipolar I or II disorder, confirmed by the Mini-International Neuropsychiatric Interview (MINI; [27]). Mild to moderate depressive symptoms were allowed, defined as a score of ≤38 points [28], [29] on the 30 item-Inventory of Depressive Symptomatology-self rating (IDS-SR; [30]). Hypomanic or manic symptoms were not allowed, defined as >7 points on the Young Mania Rating Scale (YMRS; [31]). Other exclusion criteria were: mental retardation (IQ<70), a known systemic or neurological disease which could influence cognitive functioning, endocrine disorders, the use of corticosteroids and the need for current treatment for substance use disorders in a specialized setting.

**Table 5:** Associations between HPA categories and depressive symptoms.

	CAR (n = 61)	Diurnal (n = 62)	Evening (n = 58)	DST (n = 56)
	Beta† [95%CI]	Beta† [95%CI]	Beta† [95%CI]	Beta† [95%CI]
IDS-SR	−0.02 [−0.05; 0.02]	0.39 [0.02; 0.77]	−0.01 [−0.09; 0.06]	−0.03[−0.12; 0.06]

† All values are regression coefficients (beta's) indicating the mean change in IDS-SR total score, divided by 13, associated with an increase of 1 unit HPA value.

Higher values indicate worse performance.

Betas are corrected for age, gender, education and IQ.

IDS-SR = Inventory of Depressive Symptomatology-self rating; CAR = cortisol awakening response; Diurnal = diurnal slope; evening = mean evening cortisol level; DST = cortisol suppression on the dexamethasone suppression test; 95%CI = 95% confidence interval.

All patients who took part in the larger study were asked to participate in the current study which implied saliva sampling within 4 weeks after completion of the cognitive tests. Additional exclusion criteria were: current pregnancy, current breastfeeding, current use of corticosteroids or a delay of more than 30 days between cognitive testing and cortisol sampling. Due to refusal to take part in this additional study (n = 30, 27.3%), the use of corticosteroids (n = 2, 1.8%), missing saliva data (n = 6, 5.5%) and delay of more than 30 days of saliva sampling (n = 7, 6.4%), 65 out of 110 (59.1%) bipolar patients participated in the present study. Non-participants did not differ from the final bipolar sample in age (t = −1.39, p = 0.17), education level (t = −0.41, p = 0.69), gender (χ^2^ = 3.44, p = 0.06) or subtype of bipolar disorder (χ^2^ = 0.75, p = 0.69).

**Table 6:** The explaining effect of diurnal slope (n = 62) on the associations between depressive symptoms and cognitive performance (only beta's presented).

	Beta† [95%CI]	Beta£ [95%CI]	% change [95% CI]
Speed	0.47 [0.14; 0.79]	0.50 [0.16; 0.84]	6.76 [−9.35; 65.58]
Process	0.26 [−0.10; 0.61]	0.26 [−0.11; 0.63]	1.21 [−40.36; 40.16]
Attention	0.26 [0.04; 0.49]	0.29 [0.06; 0.53]	12.22 [−1.49; 59.68]
Mean	0.21 [0.04; 0.37]	0.24 [0.07; 0.41]	16.71 [−1.90; 68.14]

For all measures: Beta's are corrected for age, gender, education and IQ.

Higher values indicate worse performance.

95%CI: 95% confidence interval.

† Regression coefficients (beta's) indicating the mean change in cognitive performance, associated with an increase of 13 points IDS-SR score.

£ Regression coefficients (beta's), after inclusion of diurnal slope in the model.

Diurnal = diurnal slope; Speed = psychomotor speed; Process = speed of information processing; Attention = attentional switching; Mean = mean z-score of all 6 cognitive domains; % change = difference score divided by startvalue ^†^; 95%CI = 95% confidence interval.

### Measurements

#### Measurements of characteristics and symptoms

Clinical evaluation took place during the phase of cognitive testing. Patients' and lifetime illness characteristics were provided by the clinician via the Questionnaire for Bipolar Disorder (BP; an adaption of the Enrollment Questionnaire as previously used in the Stanley Foundation Bipolar Network; [32], [33]). In case of mismatch of results from the MINI in relation to the QBP, diagnoses were checked with the treating clinician. The level of depressive symptoms was measured by the 30 item-Inventory of Depressive Symptomatology-self rating [IDS-SR; [30]]. The level of education was based on the Dutch educational system which differentiates already after primary school into different levels, ranging from 1: primary school up to 6: PhD student or higher degree.

#### Neurocognitive assessment

The cognitive test battery has been described elsewhere [26]. In short, the composition of the cognitive test battery was based on existing literature and experience with the target group in clinical practice. The battery included six cognitive domains (psychomotor speed, speed of information processing, attentional switching, verbal memory, visual memory and executive functioning/working memory), consisting of seven different tests, yielding 14 outcome variables (see Table 1). In addition, premorbid intelligence (IQ) was estimated with the National Adult Reading Test (NART; [34]). Detailed descriptions of the pen-and-paper measures are provided by Lezak et al. [35]. Robbins et al. [36] discussed the tests from the Cambridge Neuropsychological Test Automated Battery, a computer-based cognitive assessment system which can be administered to subjects using a touch screen computer to examine various areas of cognitive function. The total cognitive test battery was administered within about 2½ hours, with one break if necessary.

#### Cortisol analyses

Saliva cortisol sampling was performed according to procedures of the Netherlands Study of Depression and Anxiety (NESDA; [11], [37]). Salivary sampling has shown to be a reliable and minimally intrusive method to assess the active, unbound form of cortisol [38]. Patients were instructed to collect saliva samples at a day with regular activities (not a resting day, e.g. weekend or holiday) as soon as possible after the cognitive testing day. The median time between the cognitive testing day and saliva sampling was 6.0 days (25^th^–75^th^ percentile, 2.0–11.5 days). Together with the saliva samples patients filled out a questionnaire, which described the time of awakening and subsequent saliva sampling times with the first one at awakening (T1), 30 (T2), 45 (T3) and 60 (T4) minutes after awakening and evening levels at 10 pm (T5) and 11 pm (T6).

Also in accordance with NESDA [11], [37], four cortisol indicators were used in this study: cortisol awakening response (CAR), evening cortisol level, diurnal slope, and cortisol suppression after the administration of 0.5 mg dexamethasone at 2300 h (T6; dexamethasone suppression test; DST). The CAR included four sampling points: at awakening (T1) and at 30 (T2), 45 (T3) and 60 (T4) minutes later. The CAR was assessed by calculating the area under the curve (AUC) with respect to the ground (AUCg) and with respect to the increase (AUCi), conform the formulas by Pruessner et al. [39]. Only the AUCg was used for further analysis as the AUCi was in 25 cases negative due to the fact that T1 was higher than the subsequent values after awakening. Values were assigned missing in case of sampling outside a margin of 5 minutes before or after the time protocol or in case of more than 2 SDs from the mean. For AUC analyses, at least 3 samples had to be available. In 10 cases the missing value was imputed using linear regression analyses including information on the available 3 cortisol levels, sex, age and awakening time. Evening cortisol levels were collected at 10 pm (T5) and 11 pm (T6). The mean of the two evening levels (T5 and T6) was used for further analyses of evening cortisol, since both values were moderately correlated (Pearson R = 0.41, p = 0.002). Diurnal slope was calculated by subtracting the value at 2300 h (T6) from the value at awakening (T1) and dividing it by the number of hours in between the two samples, resulting in the decline in cortisol level per hour [40]. The cortisol suppression ratio in the DST was calculated by dividing the cortisol value at awakening on the first day (T1) by the cortisol value at awakening the next morning (T7) by strict instructions after the patients had taken the dexamethasone. The sample of the current analyses consisted of 61 out of 65 (93.8%) patients for CAR-analyses, 58 (89.2%) patients for evening cortisol-analyses, 62 (95.4%) patients for diurnal slope-analyses and 56 (86.2%) patient for DST-analyses. Comparing the cortisol values with previous studies regarding unipolar and bipolar depressed patients versus control groups, the cortisol data in this study are in the same range [11], [14].

### Statistical analyses

Cognitive test scores were obtained in a previous study in which we included 110 bipolar patients [26]. In line with this study cognitive scores were transformed into age-adjusted z-scores using the mean and standard deviations of 75 healthy controls. Higher scores indicated worse performance. Cognitive domain scores were created by calculating the mean z-value of the pertaining cognitive variables. In addition, a mean score was created by averaging the z-scores of all six domains.

The association of depressive symptoms with cognitive functioning was quantified by performing linear regression analyses with the age-corrected z-scores for cognition as the dependent variable, and the IDS-SR total score and potential confounders for this association gender, education and IQ as independent variables. The continuous IDS-SR total score was divided by 13 and consequently the beta's reported are per 13 points on the IDS-SR. The choice of 13 points is essentially arbitrary but approximately corresponds to continuously shifting from the level of none (0–13) to mild (14–25) or from mild to moderate (25–38) depressive symptoms, irrespective of the starting level of depressive symptoms [28], [29].

The associations of HPA axis activity indicators with cognitive functioning and depressive symptoms were performed using the same approach. In these analyses, higher scores of the HPA indicators meant worse functioning, indicating a higher rate of HPA axis dysregulation.

The last step was to investigate the potential explanation of the associations between depressive symptoms and cognitive functioning by HPA-axis functioning. The amount of explanation was defined as the change in the magnitude of the relation between depressive symptoms and cognitive functioning when including the HPA axis indicator as an independent variable. It was scaled as the relative change in the regression coefficient for the HPA indicator and was expressed as a percentage. We applied bootstrapping techniques drawing 5000 samples with replacement to obtain 95% confidence intervals (95%CI) for the percentages explained [41]. The analyses of percentage explained were restricted to those cognitive domains shown to be significantly related to depressive symptoms in our prior study [26] and to those HPA axis indicators showing a statistically significant association with depressive symptoms or with cognitive performance. The linear regression model assumptions of normality, linearity and homoscedasticity were assessed using residual plots and were found to be sufficiently met in all analyses.

The level of statistical significance was set at 0.05, two-sided. Analyses were performed using SPSS Version 16.0 [42].

## Results

### Sample characteristics

Raw neurocognitive data are presented in Table 1. Demographic and clinical characteristics of the bipolar sample are listed in Table 2. The study included 53 bipolar I patients (81.5%) and 12 bipolar II patients (18.5%). Patients were euthymic [n = 29; IDS-SR score <14 [28], [29]], mildly depressed [n = 19; IDS-SR score 14–25 [28], [29]] or moderately depressed [n = 17; IDS-SR score 26–38 [28], [29]] with a mean IDS-SR score of 17.0 (SD = 10.7). Only two bipolar patients (3.1%) were medication-free; the majority of all other patients used 1 (52.3%, n = 34) psychotropic drug. Unfortunately, no complete information on dosage of psychotropic medication is available.

### Association between depressive symptoms and cognitive functioning

Evaluation within subgroups of availability of the respective HPA axis indicator showed that depressive symptoms were modestly associated with psychomotor speed, attentional switching and the mean cognitive score; effect sizes were in the range of 0.19 and 0.50 (see Table 3). These effect sizes were comparable with our prior study in the total sample [26].

### Association between HPA axis activity and cognitive functioning

Table 4 demonstrates that changes in HPA axis activity were not associated with any of the cognitive domains.

### Association between HPA axis activity and depressive symptoms

From Table 5 it can be seen that depressive symptoms were modestly associated with attenuation in diurnal slope value only.

### The potentially explaining role of HPA axis activity in the association between depressive symptoms and cognitive functioning

Table 6 presents the results of the analyses on the explanation of the association of depressive symptoms with cognition by diurnal slope, since this HPA axis indicator was the only one that was shown to be associated with either cognitive functioning or depressive symptoms (Tables 4 and 5). After addition of this HPA axis indicator to the independent variables as a possibly explaining factor, only minute and statistically non-significant mediated effects were found in the percentage change-range of 1% to 17% with large 95%-confidence intervals, all including zero.

## Discussion

In our previous study (van der Werf-Eldering et al., 2010) we showed that cognitive dysfunction is associated with depressive symptoms in bipolar disorder. The aim of the current study was to investigate a potential mechanism explaining this relationship. Variation in HPA-axis activity, and therewith altered circulating cortisol levels, might play a role as disturbances of the HPA axis have been reported in bipolar disorder and depression [8]–[13], [15] and to be associated with cognitive disturbances in both healthy subjects and subjects with mood disorders [18]–[20], [23]–[25].

In the present study we assessed the magnitude by which the association between depressive symptoms and cognitive function is explained by HPA-indicators. The amount of explanation appeared negligible and therefore we could not confirm the hypothesis that the relationship between depressive symptoms and cognitive functioning is mediated by HPA-axis activity.

This led us to reevaluate the literature on findings supporting the hypothesis that there is a relationship between HPA-axis functioning and cognition, since negative effects of glucocorticoids on hippocampal neurons have been described in both animal experiments and humans in relation to depression [43]. Although hippocampal atrophy is found in both hypercortisolemic patients suffering from Cushing's disease [44] and in patients with major depressive disorder [45], there is so far no evidence that this explains disturbances in cognitive functioning in depression. Stress has an effect on all memory related processes – encoding, consolidation, retrieval, and re-consolidation [46]. However, we did not find an association between depressive symptoms and the cognitive domain memory. The association between depression and cognition we found was with attentional switching and psychomotor speed. The relation between cortisol and these two domains remains unclear and has to our knowledge not specifically been studied in relation to the HPA-axis. In a study by Comijs et al. [47] the association found between levels of cortisol and domains of memory and information processing speed were independently from depression status, also suggesting that although both cognitive dysfunction and HPA-axis dysregulation co-occur in depression, the mechanism behind cognitive dysfunction might be a different one than HPA-axis dysregulation.

This study is, to our knowledge, the first investigating the relationship between HPA-axis activity and cognitive functioning in bipolar patients. We consequently compared our results with three previous studies in unipolar rather than bipolar depression keeping this limitation in mind. In unipolar depressed patients Egeland et al. [24] found an association between high morning cortisol levels and the cognitive domains executive functioning and post-encoding memory, both retrieval and storage. The 26 subjects were on average severely depressed (mean Hamilton Depression Rating Scale scores 21.4±2.9) which is in contrast with our group of 65 mildly depressed subjects (mean IDS-SR 17.0±10.7), closer to non-depressed subjects. Reppermund et al. [23] and Zobel [25] studied the relationship between the HPA axis, cognitive functioning and depression in a different way: by repeated assessments and in relation with treatment outcome. Reppermund et al. [23] reported a dissociation between depressive symptomatology and HPA-axis functioning in their impact on cognitive functioning. Zobel et al. [25] found that the reduction of the HPA axis activity was related with improvement in working memory, while this was independent of a reduction in depression severity. It is important to realize that the core hypotheses of the study of Zobel et al. was to postulate specific relationships between changes in three formulated domains of correlates of depressive disorders, to mention the severity of depressive symptoms, cognitive measures and HPA responsivity to the DEX/CRH test using repeated measurements over a period of 4 weeks; our study was pointed towards another question, investigating the potential role of HPA axis dysfunction in order to explain the relationship between cognitive dysfunction and depressive symptoms. Besides different diagnoses (major depressive disorder versus bipolar disorder), the inclusion criteria with regard to the severity of depressive symptoms was different; Zobel et al. accepted only moderate-severely depressed inpatients (Hamilton Rating Scale for Depression with 17 items (HAMD17) above 18, comparable with IDS-SR score above 36) with the average of HAMD17 of 25 (comparable with IDS-SR score above 48). Our study excluded patients with IDS-SR score above 39. It is likely to assume that the higher the level of depressive symptoms, the more fluctuations in HPA axis functioning could be observed. Zobel et al. also made use of the DEX/CRH test, known to be more sensitive than the dexametasone suppression test. However, our cognitive test battery included a variety of tests, leading to an average score of each domain, probably reflecting a more realistic picture of cognitive functioning instead of calculating with the outcome scores of one single test with the risk of learning effect by repeated measurements, as was done in the study of Zobel et al.

To appreciate our findings some strengths and limitations have to be mentioned. With respect to the reliability of our data assessment, the cognitive test battery used in our study covers all relevant cognitive domains with regard to bipolar disorder, but by averaging different tasks within the corresponding domains, it reduces multiple comparisons for data analysis and avoids disproportionate emphasis of single test results [48]. In this manner we expanded the number of domains that have been evaluated in depressed bipolar patients [2]. Regarding the HPA axis we used the same, well defined parameters and protocol for assessing the HPA-axis responsiveness (DST and CAR) and cortisol levels (diurnal slope and evening cortisol levels) as used in the NESDA study [11], [14], [37]. However, despite the overall quality of the HPA-indicators, the reliability of the DST is limited, due to the fact that only one sample was taken at awakening the morning after dexamethasone intake without later samples. In this manner the suppression of the rise of cortisol after awakening may have been missed. Also, the time lapse between neuropsychological assessment and saliva sampling in our study with a median of 6 days could be a possible limitation. As the HPA-system is very sensitive for stressful events it is possible that in some cases the HPA-axis indicators might have changed compared to the moment of neuropsychological testing. Also, due to lack of available data, the effect of smoking and clinical determinants (e.g. duration of illness) regarding HPA-axis indicators could not be evaluated. Another potential limitation is the fact that exclusion of possible dementia was made clinically without formal assessments. In addition, the sample size of our study counted a total number of 65 bipolar patients. Although this number is limited regarding evaluation of more detailed analyses, it seemed to be sufficient to answer the primary question. The percentages explained were mostly close to zero and it is in our view unlikely that they would become statistically significant if we had included a larger sample. Finally, our study was cross-sectional which leaves the temporal order of events unspecified. Consequently, we could not distinguish between on the one hand a model in which HPA axis disturbances causes depressive symptoms which in turn cause cognitive problems (through another mechanism), and on the other hand a model in which HPA axis disturbances cause both depressive symptoms and cognitive deficits, i.e. they form a common cause.

In conclusion, in line with previous studies in unipolar depression, our results indicate that the significant association between severity of depressive symptoms and cognitive functioning in bipolar patients cannot be explained by changes in HPA-axis activity. As our study is the first one in this field specific for bipolar patients, other studies are needed to evaluate other factors that might explain the relationship between depressive symptoms and cognitive functioning. Future studies on mediation of the relationship between cognitive dysfunction and mood symptoms should preferably be longitudinal. In addition, more knowledge is needed and recent research in psychiatric disorders hypothesizes for example about inefficient glucocorticoid signaling at different levels [21], [49], but also about other divergent kinds of knowledge, like a link with inflammatory processes [50], with sleep [51], or with cerebrovascular and degenerative changes [52].

## References

[1] McDermott LM, Ebmeier KP (2009). A meta-analysis of depression severity and cognitive function.. J Affect Disord.

[2] Kurtz MM, Gerraty RT (2009). A meta-analytic investigation of neurocognitive deficits in bipolar illness: Profile and effects of clinical state.. Neuropsychology.

[3] Torres IJ, Boudreau VG, Yatham LN (2007). Neuropsychological functioning in euthymic bipolar disorder: A meta-analysis.. Acta Psychiatr.

[4] Robinson LJ, Thompson JM, Gallagher P, Goswami U, Young AH (2006). A meta-analysis of cognitive deficits in euthymic patients with bipolar disorder.. J Affect Disord.

[5] Watson S, Gallagher P, Ritchie JC, Ferrier IN, Young AH (2004). Hypothalamic-pituitary-adrenal axis function in patients with bipolar disorder.. Br J Psychiatry.

[6] Gallagher P, Malik N, Newham J, Young AH, Ferrier IN (2008). Antiglucocorticoid treatments for mood disorders..

[7] Spijker AT, van Rossum EF (2009). Glucocorticoid receptor polymorphisms in major depression. focus on glucocorticoid sensitivity and neurocognitive functioning.. Ann N Y Acad Sci.

[8] Heuser I, Yassouridis A, Holsboer F (1994). The combined dexamethasone/CRH test: A refined laboratory test for psychiatric disorders.. J Psychiatr Res.

[9] Holsboer F (2000). The corticosteroid receptor hypothesis of depression.. Neuropsychopharmacology.

[10] Carroll BJ, Feinberg M, Greden JF, Tarika J, Albala AA (1981). A specific laboratory test for the diagnosis of melancholia. standardization, validation, and clinical utility.. Arch Gen Psychiatry.

[11] Vreeburg SA, Hoogendijk WJ, van Pelt J, Derijk RH, Verhagen JC (2009). Major depressive disorder and hypothalamic-pituitary-adrenal axis activity: Results from a large cohort study.. Arch Gen Psychiatry.

[12] Deshauer D, Duffy A, Alda M, Grof E, Albuquerque J (2003). The cortisol awakening response in bipolar illness: A pilot study.. Can J Psychiatry.

[13] Daban C, Vieta E, Mackin P, Young AH (2005). Hypothalamic-pituitary-adrenal axis and bipolar disorder.. Psychiatr Clin North Am.

[14] Jabben N, Nolen WA, Smit JH, Vreeburg SA, Beekman AT (2011). Co-occurring manic symptomatology influences HPA axis alterations in depression.. J Psychiatr Res.

[15] Porter RJ, Gallagher P (2006). Abnormalities of the HPA axis in affective disorders: Clinical subtypes and potential treatments.. Acta Neuropsychiatrica.

[16] Rush AJ, Giles DE, Schlesser MA, Orsulak PJ, Parker CR (1996). The dexamethasone suppression test in patients with mood disorders.. J Clin Psychiatry.

[17] McQuade R, Young AH (2000). Future therapeutic targets in mood disorders: The glucocorticoid receptor.. Br J Psychiatry.

[18] Lupien SJ, McEwen BS (1997). The acute effects of corticosteroids on cognition: Integration of animal and human model studies.. Brain Res Brain Res Rev.

[19] Liston C, McEwen BS, Casey BJ (2009). Psychosocial stress reversibly disrupts prefrontal processing and attentional control.. Proc Natl Acad Sci U S A.

[20] Beluche I, Carriere I, Ritchie K, Ancelin ML (2010). A prospective study of diurnal cortisol and cognitive function in community-dwelling elderly people.. Psychol Med.

[21] Brown ES (2009). Effects of glucocorticoids on mood, memory, and the hippocampus. treatment and preventive therapy.. Ann N Y Acad Sci.

[22] Brown ES, Rush AJ, McEwen BS (1999). Hippocampal remodeling and damage by corticosteroids: Implications for mood disorders.. Neuropsychopharmacology.

[23] Reppermund S, Zihl J, Lucae S, Horstmann S, Kloiber S (2007). Persistent cognitive impairment in depression: The role of psychopathology and altered hypothalamic-pituitary-adrenocortical (HPA) system regulation.. Biol Psychiatry.

[24] Egeland J, Lund A, Landro NI, Rund BR, Sundet K (2005). Cortisol level predicts executive and memory function in depression, symptom level predicts psychomotor speed.. Acta Psychiatr Scand.

[25] Zobel AW, Schulze-Rauschenbach S, von Widdern OC, Metten M, Freymann N (2004). Improvement of working but not declarative memory is correlated with HPA normalization during antidepressant treatment.. J Psychiatr Res.

[26] van der Werf-Eldering MJ, Burger H, Holthausen EAE, Aleman A, Nolen WA (2010). Cognitive functioning in patients with bipolar disorder: Association with depressive symptoms and alcohol use.. PloS ONE.

[27] Sheehan DV, Lecrubier Y, Sheehan KH, Amorim P, Janavs J (1998). The mini-international neuropsychiatric interview (M.I.N.I.): The development and validation of a structured diagnostic psychiatric interview for DSM-IV and ICD-10.. J Clin Psychiatry.

[28] Trivedi MH, Rush AJ, Ibrahim HM, Carmody TJ, Biggs MM (2004). The inventory of depressive symptomatology, clinician rating (IDS-C) and self-report (IDS-SR), and the quick inventory of depressive symptomatology, clinician rating (QIDS-C) and self-report (QIDS-SR) in public sector patients with mood disorders: A psychometric evaluation.. Psychol Med.

[29] Rush AJ, Trivedi MH, Ibrahim HM, Carmody TJ, Arnow B (2003). The 16-item quick inventory of depressive symptomatology (QIDS), clinician rating (QIDS-C), and self-report (QIDS-SR): A psychometric evaluation in patients with chronic major depression.. Biol Psychiatry.

[30] Rush AJ, Gullion CM, Basco MR, Jarrett RB, Trivedi MH (1996). The inventory of depressive symptomatology (IDS): Psychometric properties.. Psychol Med.

[31] Young RC, Biggs JT, Ziegler VE, Meyer DA (1978). A rating scale for mania: Reliability, validity and sensitivity.. Br J Psychiatry.

[32] Suppes T, Leverich GS, Keck PE, Nolen WA, Denicoff KD (2001). The stanley foundation bipolar treatment outcome network. II. demographics and illness characteristics of the first 261 patients.. J Affect Disord.

[33] Leverich GS, Nolen WA, Rush AJ, McElroy SL, Keck PE (2001). The stanley foundation bipolar treatment outcome network. I. longitudinal methodology.. J Affect Disord.

[34] Nelson HE (1982). National adult reading test (NART) test manual..

[35] Lezak MD, Howieson DB, Loring DW (2004). Neuropsychological assessment..

[36] Robbins TW, James M, Owen AM, Sahakian BJ, McInnes L (1994). Cambridge neuropsychological test automated battery (CANTAB): A factor analytic study of a large sample of normal elderly volunteers.. Dementia.

[37] Vreeburg SA, Kruijtzer BP, van Pelt J, van Dyck R, DeRijk RH (2009). Associations between sociodemographic, sampling and health factors and various salivary cortisol indicators in a large sample without psychopathology.. Psychoneuroendocrinology.

[38] Kirschbaum C, Hellhammer DH (1994). Salivary cortisol in psychoneuroendocrine research: Recent developments and applications.. Psychoneuroendocrinology.

[39] Pruessner JC, Kirschbaum C, Meinlschmid G, Hellhammer DH (2003). Two formulas for computation of the area under the curve represent measures of total hormone concentration versus time-dependent change.. Psychoneuroendocrinology.

[40] Bhattacharyya MR, Molloy GJ, Steptoe A (2008). Depression is associated with flatter cortisol rhythms in patients with coronary artery disease.. J Psychosom Res.

[41] Preacher KJ, Hayes AF (2008). Asymptotic and resampling strategies for assessing and comparing indirect effects in multiple mediator models.. Behav Res Methods.

[42] Statistical Package for the Social Sciences (SPSS) 1602forWindows (2008). SPSS Inc..

[43] Sapolsky RM (2000). Glucocorticoids and hippocampal atrophy in neuropsychiatric disorders.. Arch Gen Psychiatry.

[44] Starkman MN, Gebarski SS, Berent S, Schteingart DE (1992). Hippocampal formation volume, memory dysfunction, and cortisol levels in patients with cushing's syndrome.. Biol Psychiatry.

[45] Sheline YI (1996). Hippocampal atrophy in major depression: A result of depression-induced neurotoxicity?. Mol Psychiatry.

[46] Schwabe L, Joels M, Roozendaal B, Wolf OT, Oitzl MS (2011). Stress effects on memory: An update and integration..

[47] Comijs HC, Gerritsen L, Penninx BW, Bremmer MA, Deeg DJ (2010). The association between serum cortisol and cognitive decline in older persons.. Am J Geriatr Psychiatry.

[48] Keefe RS (1995). The contribution of neuropsychology to psychiatry.. Am J Psychiatry 152(0002-953;.

[49] Aas M, Dazzan P, Mondelli V, Toulopoulou T, Reichenberg A (2011). Abnormal cortisol awakening response predicts worse cognitive function in patients with first-episode psychosis.. Psychol Med.

[50] Rohleder N, Wolf JM, Wolf OT (2010). Glucocorticoid sensitivity of cognitive and inflammatory processes in depression and posttraumatic stress disorder.. Neurosci Biobehav Rev.

[51] Altena E, Van Der Werf YD, Strijers RL, Van Someren EJ (2008). Sleep loss affects vigilance: Effects of chronic insomnia and sleep therapy.. J Sleep Res.

[52] Grool AM, van der Graaf Y, Mali WP, Geerlings MI, SMART Study Group. (2011). Location of cerebrovascular and degenerative changes, depressive symptoms and cognitive functioning in later life: The SMART-medea study.. J Neurol Neurosurg Psychiatry.

